# VRD versus VCD as induction therapy before autologous stem cell transplantation in multiple myeloma: a nationwide population-based study

**DOI:** 10.1038/s41408-024-01047-1

**Published:** 2024-04-09

**Authors:** Jakob Nordberg Nørgaard, Kari Lenita Falck Moore, Tobias S. Slørdahl, Anders Vik, Tor Henrik Anderson Tvedt, Fredrik Schjesvold

**Affiliations:** 1 Oslo Myeloma Center, Department of Hematology, Oslo University Hospital, Oslo, Norway; 2 Institute of Clinical Medicine, University of Oslo, Oslo, Norway; 3 KG Jebsen Center for B cell malignancies, University of Oslo, Oslo, Norway; 4 Department of Hematology and Oncology, Stavanger University Hospital, Stavanger, Norway; 5 Department of Clinical and Molecular Medicine, Norwegian University of Science and Technology (NTNU), Trondheim, Norway; 6 Department of Hematology, St. Olavs hospital, Trondheim University Hospital, Trondheim, Norway; 7 Department of Hematology, University Hospital of North Norway, Tromsø, Norway; 8 Institute of Clinical Medicine, The Arctic University of Norway, UiT, Tromsø, Norway; 9 Department of Hematology, Oslo University Hospital, Oslo, Norway; 10 Clinic for Medicine, Haukeland University Hospital, Bergen, Norway

**Keywords:** Epidemiology, Myeloma

Induction therapy followed by autologous stem cell transplantation (ASCT) is standard of care for young and fit patients with newly diagnosed multiple myeloma (MM) [1]. Induction therapy has evolved from doublet to triplet to quadruplet regimens over the last decades. The most common triplet therapy is either Bortezomib-Cyclophosphamide-Dexamethasone (VCD), Bortezomib-Lenalidomide-Dexamethasone (VRD), or less frequently Bortezomib-Thalidomide-Dexamethasone (VTD). No large, randomized phase III study comparing the VCD and VRD regimens has been conducted and is unlikely to be done in the future. Retrospective studies and smaller prospective studies comparing VRD and VCD have produced mixed results [2–7].

In Norway, there has been a shift from VCD to VRD induction therapy in recent years, while VTD has only been used in a minority of patients. ASCT for multiple myeloma in Norway is performed at four centers, and comprehensive population-based nationwide follow-up data are available from electronic journals.

In collaboration with all centers in Norway doing ASCT, we identified all patients in Norway who had undergone ASCT for multiple myeloma in the study period 2008 to 2020.

We included patients with multiple myeloma [8] who received first line induction therapy followed by ASCT in the period from January 1^st^ 2008 to December 31^st^ 2020 in Norway. We did not include patients who received induction therapy but did not proceed to ASCT. Patients were censored March 1^st^ 2022 or at loss to follow-up because of relocation outside of Norway (*n* = 5), or if the journal from the local hospital could not be obtained (*n* = 7).

Data was collected from electronic patient journals at the transplant centers and from hospitals responsible for induction therapy and follow-up after ASCT. Change of induction therapy was recorded if a patient changed from one line to another, and the reason for change was collected. Patients who changed therapy were not included in the primary response analysis, regardless of the reason for change, but they were included in a separate intention-to-treat analysis. All patients, including those who changed treatment, were included in the PFS and OS analysis. Further description of study design, endpoints and statistical analysis is provided in the supplementary material.

We identified 1354 patients who received ASCT as first-line treatment for multiple myeloma in Norway in the study period.

Of these, 682 patients received VCD induction, 332 patients received VRD induction, and 42 patients received VTD induction. Baseline characteristics are described in Table 1 and were largely similar between patients who received VCD and VRD induction, with two notable exceptions. Patients in the VRD group were older than patients in the VCD group (median 62 years vs. 60 years). Patients in the VRD group received ASCT in more recent years (mostly 2017-2020), compared to VCD.

**Table 1:** Baseline characteristics, response rates, reason for change of therapy and treatment administered after ASCT according to induction treatment.

	VCD induction (*n* = 682)	VRD induction (*n* = 332)	*p* value
**Median age at ASCT, years (range)**	60 (30–71)	62 (30–75)	<0.001
**Male sex**	416 (61%)	193 (58%)	0.382
**Diagnostic criteria**^**a**^			
Kidney failure	83 (12%)	32 (10%)	0.239
Anemia	344 (50%)	161 (48%)	0.561
Osteolytic lesion(s)	547 (80%)	265 (80%)	0.885
Hypercalcemia	102 (15%)	40 (12%)	0.215
SLiM only^b^	18 (3%)	21 (6%)	0.004
**Year of ASCT**			
2008-2013	256 (38%)	0 (0%)	
2014-2016	255 (37%)	1 (0%)	
2017-2020	171 (25%)	331 (100%)	
**ISS stage**			0.933
ISS I and II:	409 (76%)	228 (86%)	
ISS III:	131 (24%)	72 (24%)	
Missing	142	32	
**R-ISS stage**			0.805
R-ISS I and II:	269 (89%)	228 (89%)	
R-ISS III:	32 (11%)	29 (11%)	
Missing	381	75	
**Cytogenetic risk profile**			0.561
Standard	265 (81%)	221 (79%)	
High-risk^c^	64 (19%)	60 (21%)	
Missing	353	51	
**Response rates**			
≥VGPR response pre-ASCT	283 (49%)	226 (74%)	<0.001
≥VGPR response 3 months after ASCT	453 (76%)	279 (89%)	<0.001
Death at 3 months after ASCT	3 (1%)	1 (0%)	
**Intention-to-treat analysis**			
≥VGPR pre-transplant	295 (47%)	235 (73%)	<0.001
≥VGPR 3 months after ASCT	477 (74%)	292 (88%)	<0.001
**Change of therapy**	49 (7%)	17 (5%)	0.215
Lack of response	24 (4%)	2 (1%)	
Side effects	16 (2%)	11 (3%)	
Progression	6 (1%)	4 (1%)	
Doctor’s choice	3 (0%)	0 (0%)	
**Post-ASCT treatment**			
None	586 (86%)	129 (39%)	<0.001
Consolidation treatment only	6 (1%)	74 (22%)	
Maintenance treatment only	66 (10%)	83 (25%)	
Both consolidation and maintenance	18 (3%)	45 (14%)	
**Consolidation therapy**			
VRd	8 (1.2%)	60 (18%)	
KRd	7 (1.0%)	31 (9.3%)	
Rd	4 (0.6%)	27 (8.1%)	
Other	4 (0.6%)	2 (0.6%)	
**Maintenance therapy**			
Lenalidomide	66 (10%)	121 (32%)	
Bortezomib	4 (0.6%)	6 (1.8%)	
Ixazomib	9 (1.3%)	0 (0%)	
Other	4 (0.6%)	2 (0.6%)	
**Duration of maintenance**^**d**^			0.502
≥18 months	50 (62%)	77 (66%)	
<18 months:	31 (38%)	39 (34%)	

*VCD* Bortezomib, Cyclophosphamide, Dexamethasone, *VRD* Bortezomib, Lenalidomide, Dexamethasone, *KRd* Carfilzomib, Lenalidomide, Dexamethasone, *Rd* Lenalidomide, Dexamethasone, *ASCT* Autologous stem cell transplantation, *VGPR* Very good partial response, *ISS* International staging system, *R*-*ISS* Revised international staging system.

^a^Kidney failure: Creatinine > 177 μmol/L or CrCl <40 mL/min. Anemia: Hemoglobin <10 g/dL or >2 g/dL below normal. Osteolytic lesions: one or more osteolytic lesion detected by X-ray, CT or PET-CT. Hypercalcemia: Hypercalcemia: serum calcium >0.25 mmol/L ( > 1 mg/dL) higher than the upper limit of normal or >2.75 mmol/L ( > 11 mg/dL).

^b^≥60% bone marrow plasma cells, free light chain ratio ≥100 and/or >1 MRI-defined ≥5 mm focal lesion.

^c^High risk cytogenetics was defined as (del(17p), t(4;14) or t(14;16))

VGPR = very good partial response

^d^Patients who had less than 18 months of follow-up were excluded.

Three months after ASCT, response rates were higher with VRD, with 89% in the VRD group achieving ≥VGPR, versus 76% in the VCD group (*p* < 0.001). In the intention-to-treat analysis, the difference in response between the groups remained statistically significant (Table 1).

In the VCD group, 4% of patients changed therapy due to lack of response, and 1% due to progression. In the VRD group, very few patients changed treatment due to lack of response (1%) or progression (1%). Only a small minority, 3% and 2% of patients in the VRD and VCD group respectively, changed treatment due to adverse effects (Table 1). In patients who received bortezomib, thalidomide and dexamethasone (VTD), 36% of patients changed treatment due to side effects (Supplementary Table 5).

Patients in the VRD group more often received treatment after ASCT than in the VCD group (61% vs. 14%, *p* < 0.001). Consolidation treatment (22% vs. 1%), maintenance treatment (25% vs. 10%) or both (14% vs. 3%) were all more frequent in the VRD group (Table 1). In the VCD group, 4.7% of patients (*n* = 32) and in the VRD group 3.9% of patients (*n* = 13) had progressive disease before starting consolidation or maintenance treatment.

The median follow-up time of patients still alive at data cut-off was 79 months (range: 19–179 months) in the VCD group and 38 months (range: 18–71 months) in the VRD group.

In the VCD group, the median PFS was 30.1 months (95% confidence interval (CI) 28.3–31.9 months). In the VRD group, the median PFS was 55.1 months (95% CI 46.0-not reached (NR), Fig. 1A). The difference was significant on log-rank test, *p* < 0.001. In the VTD group median PFS was 36.6 months (Supplementary Fig. 2)

**Fig. 1:**
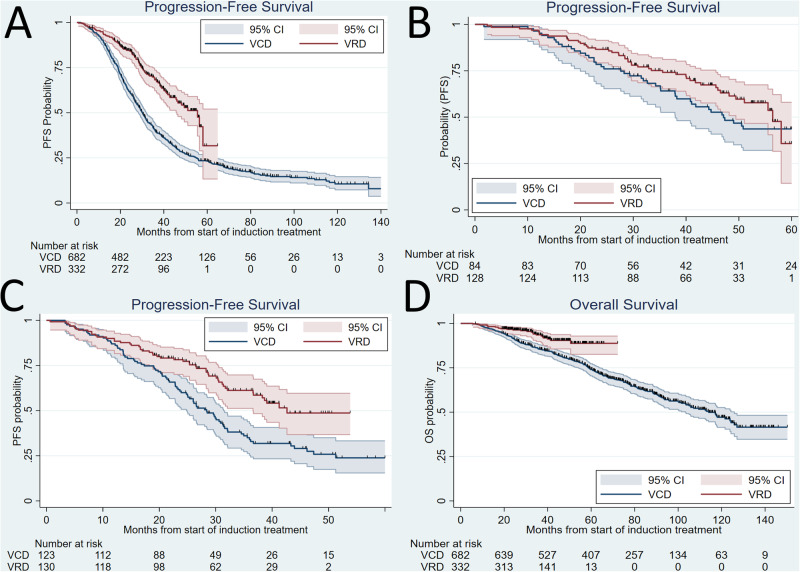
Kaplan-Meier curves according to induction treatment. **A** PFS, all patients. **B** PFS, only patients who received maintenance treatment. **C** PFS, only patients who revied ASCT in later years (2017–2020) and did not received any post-ASCT treatment. **D** OS, all patients.

When we included only patients who received maintenance therapy after ASCT, the median PFS in the VCD group increased to 47.1 months, which is not statistically different from patients in the VRD group who received maintenance, who had a median PFS of 56.4 months, *p* = 0.174 (Fig. 1B).

In a separate analysis excluding patients who received maintenance and/or consolidation and who received ASCT in later years (2017–2020), VRD was superior to VCD regarding PFS with a log-rank test of *p* < 0.001 (Fig. 1C).

The median OS for VCD was 114.0 months (95% CI 103.4–125.8 months) and the median OS for VRD was not reached, log-rank test *p* < 0.001 (Fig. 1D).

The hazard ratios for PFS and OS on multivariate analysis is provided in Supplementary Table 2. After controlling for patient and disease factors, VCD had inferior PFS compared to VRD (HR 2.08, 95% CI 1.49–2.91, *p* < 0.001). There was no significant difference in OS between the two regimens in multivariate analysis.

VTD is approved by the European Medical Agency as induction therapy before ASCT. This is not the case for VCD and VRD, although they are used widely in current clinical practice. The most recent European Society of Medical Oncology (ESMO) guidelines [1] recommend VRD as the first option for induction therapy. Daratumumab-VTD is also approved and recommended, but our study confirms the high toxicity associated with regimens containing thalidomide. VRD is the comparator arm in recent clinical trials comparing Daratumumab-VRD vs VRD before ASCT [9, 10]. Our study supports the use of VRD in both clinical practice, and as the standard treatment arm in clinical trials, as it is more effective than VCD and better tolerated than VTD. However, recent results from the PERSEUS trial [9], with significantly longer PFS for Daratumumab-VRD vs VRD, will most likely be practice changing.

We observed a statistically significant improvement in both PFS and OS favoring VRD. This must, however, be interpreted with caution. The difference in use of post-ASCT therapy, and the time periods in which the regimes were given, are two major biases. We corrected for this by performing a separate analysis for only patients who received ASCT in later years and who did not receive consolidation and/or maintenance therapy. In this analysis VRD still showed significantly longer PFS compared to VCD. The median PFS was also longer in the VRD group when only patients who received maintenance therapy were included, although the difference was not statistically significant. Multivariate analysis showed a statistically significant PFS benefit favoring VRD, but no statistically significant OS benefit. Given the median overall survival in our data of approximately 9.5 years in the VCD group, induction therapy administered for 2–4 months represents only a fraction of this total observed time. Therefore, the effect of induction therapy on overall survival may be modest, and other factors, like treatment options available at relapse, will have a significant impact on patient survival. Most patients in the VCD group received ASCT before 2017, when consolidation and maintenance treatment were uncommon and fewer treatment options were available at relapse, affecting the survival of this group negatively. Conversely, in the VRD group, most patients received ASCT after 2017. In this period, maintenance treatment was usually given (or consolidation treatment when maintenance was not reimbursed), and effective treatments like CD38-antibodies and carfilzomib were available at relapse.

The main limitation of the study is its retrospective nature, and as patients were not randomized, confounding factors cannot be excluded. However, the type of induction the patient received was mainly dependent on center and not on patient or disease factors. Standard induction therapy differed between regions, where some centers consistently used VCD while others consistently used VRD. In Norway, access to new therapies is similar for all, and national and regional treatment guidelines are usually the factors that determine choice of treatment, and to a lesser degree individual patient risk factors. Furthermore, a limitation was that we only included patients who received ASCT. Patients who died before ASCT, started induction therapy but for various reasons did not proceed to ASCT, including those who could not harvest enough stem cells, were not included. The follow-up time for VRD patients was relatively short. Patients were included from many different hospitals in Norway over a long period of time, with variable practices regarding timing of treatment start, dosing schedules, response assessment and supportive care. Although the data quality was generally good, some data was missing and incomplete.

Our study is the first to report from a comprehensive nationwide, population-based cohort with a very low proportion of patients lost to follow-up. This is a major strength, as the inclusion of a broad, heterogenous population increases the generalizability of the results and reduces the risk of selection bias. Our data included an overlap period where both regimens were given. Apart from the type of induction therapy, the treatment course between the two groups were similar; Length of induction treatment, the ASCT procedure and time to response evaluation was unchanged throughout the study period and similar for both groups.

In conclusion, our results suggests that VRD should be preferred to VCD as induction therapy for newly diagnosed MM patients who are eligible for ASCT.

### Supplementary information

Supplementary Information

## Data Availability

For original data, please contact: jaknoe@ous-hf.no.
