# Supplementary material for: VRD versus VCD as induction therapy before autologous stem cell transplantation in multiple myeloma: a nationwide population-based study
Source: Blood Cancer J. 2024 Apr 9;14(1):60. doi: 10.1038/s41408-024-01047-1 (PMC11004127; doi:10.1038/s41408-024-01047-1)
Supplement: Supplementary file 1 — Supplementary Information [file 41408_2024_1047_MOESM1_ESM.docx]

**Supplementary information, tables and figures:**

**Part 1: Supplementary information regarding methods**

**Study design**

This was an investigator-initiated, retrospective cohort-study. In collaboration with all centers in Norway doing ASCT (Oslo University Hospital (Oslo), Haukeland University Hospital (Bergen), St. Olavs Hospital - Trondheim University Hospital (Trondheim), and the University Hospital of North Norway (Tromsø)), we identified all patients in Norway who had undergone ASCT for multiple myeloma in the study period 2008 to 2020. Most patients in Norway receive induction therapy and follow-up after ASCT at their local hospital and are referred to the transplant centers only for the stem cell treatment.

The study was approved by the regional ethics committee in Norway and data protection officers at all study centers. The study was conducted in compliance with the Declaration of Helsinki. Included patients had either consented through the European Bone Marrow Transplantation Registry, or they received an information letter by mail with the possibility to withdraw from the study.

**Patients**

We included patients with multiple myeloma (11) who received first line induction therapy followed by ASCT in the period from January 1^st^ 2008 to December 31^st^ 2020 in Norway. We did not include patients who received induction therapy but did not proceed to ASCT. Patients with amyloidosis were included if they also met criteria for multiple myeloma. Patients with plasma cell leukemia were excluded. Patients were censored March 1^st^ 2022 or at loss to follow-up because of relocation outside of Norway (n=5), or if the journal from the local hospital could not be obtained (n=7).

**Study procedures**

Data was collected from electronic patient journals at the transplant centers and from hospitals responsible for induction therapy and follow-up after ASCT. Routine baseline characteristics of the patients, disease characteristics, first line treatment and response were collected for all patients. They were followed for progression, next line of treatment and OS until March 1^st^, 2022. High-risk fluorescence in situ hybridization (FISH) findings were defined according to IMWG criteria (presence of t(4;14), t(14;16) or del(17p))(12).

If a patient started the first cycle of induction with a doublet (for example Bortezomib-Dexamethasone (VD) in case of kidney failure) with a plan to add a third drug after the first cycle (for example cyclophosphamide), the induction regimen was classified according to the triplet (VCD in this example). Change of induction therapy was recorded if a patient changed from one line to another, and the reason for change was collected. We used the international guidelines for determination of prior lines in myeloma(13). Patients who changed therapy were not included in the primary response analysis, regardless of the reason for change, but they were included in a separate intention-to-treat analysis. Patients who changed treatment were included in the PFS and OS analysis.

We used standard International Myeloma Working Group (IMWG) response criteria(14). However, patients with less than 5% plasma cells in bone marrow and unmeasurable M-proteins by electrophoresis but who had not performed serum and/or urine immunofixation were classified as near CR (nCR). nCR was also used if a patient fulfilled the criteria for CR based on serum and urine results, but bone marrow assessment had not been done.

Reasons for change of therapy were classified as either progression (as defined by the IMWG-criteria(14)), unacceptable side effects, doctor’s choice or lack of response (not meeting the IMWG-criteria for progression but unsatisfactory according to the treating doctor’s judgement).

**Endpoints**

The primary endpoint in our study was the difference in response rates between VRD and VCD before ASCT and 3 months after ASCT. If response was not assessed at 3 months post-ASCT, response assessment at 2-5 months after ASCT was accepted.

The co-primary endpoint in our study was the difference between VRD, VCD and VTD in failure. We defined failure as either 1) change of induction therapy or 2) poor response (less than very good partial response (VGPR) at 3 months after ASCT).

Secondary endpoints were the comparison of PFS and OS. PFS was defined as time from start of induction treatment to progression or death. OS was defined by time from start of induction treatment to death.

**Statistical analysis**

Patient characteristics were compared by using Chi-squared tests or Fisher’s exact tests for categorical variables, and Wilcoxon rank-sum test for continuous variables. PFS and OS were estimated using the Kaplan-Meier method and compared by the log-rank test. As the treatment overlapped between 2017 and 2020 and more patients in the VRD group received maintenance and/or consolidation therapy, we performed two separate Kaplan-Meier analysis; one including only patients who received maintenance therapy and one including only patients who received ASCT between 2017 and 2020 and did not receive consolidation and/or maintenance therapy. Hazard ratios (HRs) for multivariate analyses were calculated by the Cox proportional hazards regression model. The following prognostic factors were evaluated in the multivariate analysis: induction regimen, age (≥65 years vs. <65 years), sex, transplant period (2017-2020 vs. 2014-2016 and 2008-2013), ISS-score (III vs. I and II) and FISH (high risk vs. standard risk). To correct for maintenance therapy, the Cox proportional hazard model for OS was adjusted; start of maintenance (3 months after ASCT) was set as time point zero. Statistical analyses were performed using Stata statistical software (version 17.0, StataCorp)

**Part 2: Supplementary tables and figures regarding results**

**Supplementary Figure 1: Consort diagram**

**
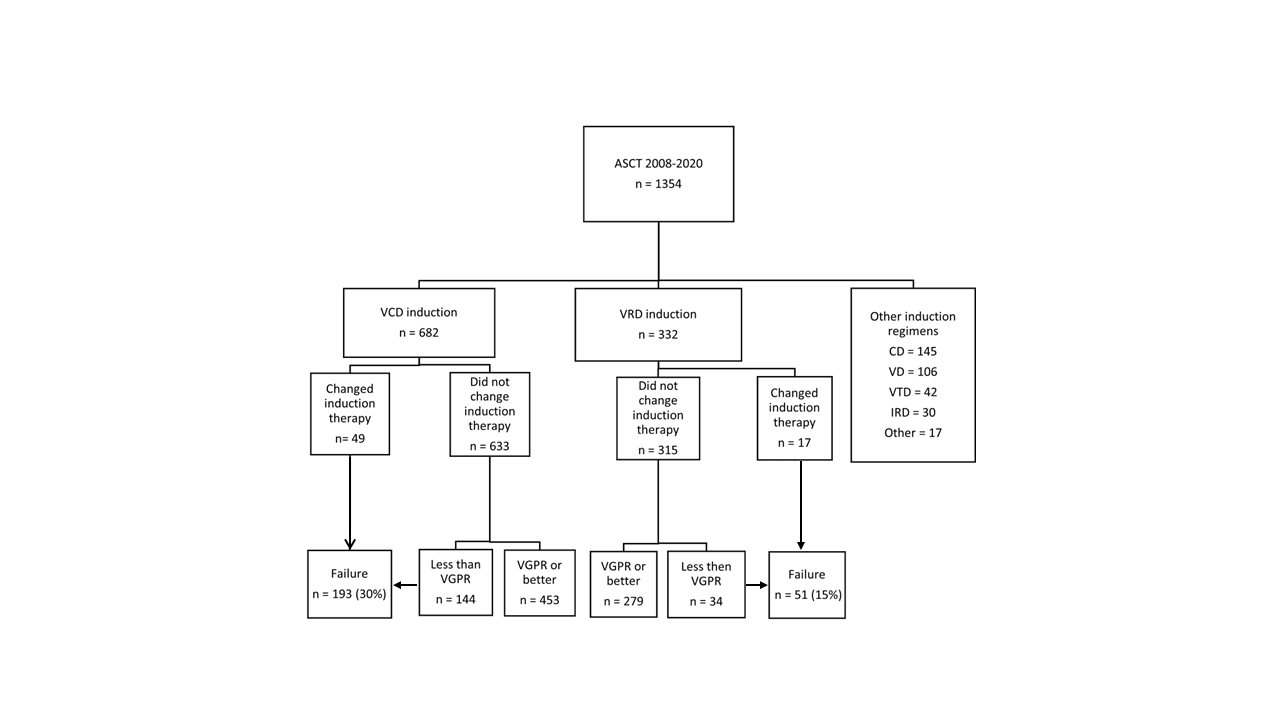
**

ASCT: autologous stem cell transplant. CD: cyclophosphamide, dexamethasone. IRD: ixazomib, lenalidomide, dexamethasone. VCD:bortezomib, cyclophosphamide, dexamethasone. VD: bortezomib, dexamethasone. VGPR: very good partial response. VRD: bortezomib, lenalidomide, dexamethasone.

**Supplementary table 1: Full response rates, VCD and VRD, pre- and post-transplant.**

|  | VCD induction | VRD induction | p-value |
| --- | --- | --- | --- |
| Pre-transplant response |  |  |  |
| nCR/CR/sCR | 92 (16%) | 79 (26%) |  |
| VGPR | 191 (33%) | 147 (48%) |  |
| ≥VGPR | 49% | 74% | <0.001 |
| PR | 247 (43%) | 76 (25%) |  |
| Overall response (≥PR) | 92% | 99% | <0.001 |
| SD | 44 (8%) | 2 (1%) |  |
| PD | 5 (1%) | 1 (0%) |  |
| Excluded due to change of therapy | 49 | 17 |  |
| Missing | 54 | 10 |  |
| Response 3 months after ASCT |  |  |  |
| nCR/CR/sCR | 210 (35%) | 162 (52%) |  |
| VGPR | 243 (41%) | 117 (37%) |  |
| ≥VGPR | 76% | 89% | <0.001 |
| PR | 111 (19%) | 27 (9%) |  |
| Overall response (≥PR) | 95% | 98% | <0.001 |
| SD | 7 (1%) | 0 (0%) |  |
| PD | 23 (4%) | 6 (2%) |  |
| Death (from any cause) | 3 (1%) | 1 (0%) |  |
| Excluded due to change of therapy | 49 | 17 |  |
| Missing | 36 | 2 |  |
| Intention-to-treat analysis |  |  |  |
| ≥VGPR pre-transplant | 295 (47%) | 235 (73%) | <0.001 |
| ≥VGPR 3 months after ASCT | 477 (74%) | 292 (88%) | <0.001 |

**Supplementary Table 2: Multivariate analysis for PFS and OS**

|  | PFS | | OS^*^ | |
| --- | --- | --- | --- | --- |
|  | **Hazard ratio** | **p-value** | **Hazard ratio** | **p-value** |
| Induction |  |  |  |  |
| VRD | Ref |  | Ref |  |
| VCD | 2.08 (1.49-2.91) | <0.001 | 1.72 (0.82-3.59) | 0.151 |
| Age |  |  |  |  |
| <65 | Ref |  | Ref |  |
| ≥65 | 0.85 (0.65-1.10) | 0.218 | 1.07 (0.67-1.72) | 0.768 |
| Sex |  |  |  |  |
| Female | Ref |  | Ref |  |
| Male | 0.99 (0.79-1.25) | 0.949 | 1.13 (0.75-1.68) | 0.562 |
| ASCT, year |  |  |  |  |
| 2017-2020 | Ref |  | Ref |  |
| 2014-2016 | 1.11 (0.80-1.53) | 0.540 | 1.00 (0.51-1.93) | 0.990 |
| 2008-2013 | 1.43 (0.98-2.10) | 0.063 | 1.77 (0.88-3.59) | 0.111 |
| ISS stage |  |  |  |  |
| I and II | Ref |  | Ref |  |
| III | 1.38 (1.06-1.79) | 0.018 | 1.63 (1.05-2.53) | 0.031 |
| Cytogenetics |  |  |  |  |
| Standard | Ref |  | Ref |  |
| High-risk | 1.43 (1.10-1.86) | 0.008 | 1.74 (1.14-2.67) | 0.011 |
| Maintenance |  |  |  |  |
| Maintenance | NA^**^ |  | Ref |  |
| No maintenance | NA^**^ |  | 1.48 (0.87-2.53) | 0.149 |

^*^OS from 3 months after ASCT (time point where patients start maintenance). Patients who died first are excluded.

^**^As these are not baseline variables, they are not included in the PFS analysis.

**Supplementary Table 3: Baseline characteristics, VTD induction and total ASCT-population**

|  | VTD induction (n=42) | All (n=1354) |
| --- | --- | --- |
| Age at ASCT |  |  |
| Median (range) | 58 (37-70) | 60 (30-75) |
| Male sex, n(%) | 25 (60%) | 807 (60%) |
| Diagnostic criteria^*^ |  |  |
| Kidney failure | 5 (12%) | 167 (12%) |
| Anemia | 17 (40%) | 672 (50%) |
| Osteolytic lesion(s) | 30 (71%) | 1075 (78%) |
| Hypercalcemia | 5 (12%) | 191 (14%) |
| SliM only^**^ | 5 (12%) | 51 (4%) |
| Year of ASCT |  |  |
| 2008-2013 | 4 | 499 |
| 2014-2016 | 30 | 298 |
| 2017-2020 | 8 | 557 |
| ISS stage n(%) |  |  |
| ISS I: | 17 (47%) | 463 (42%) |
| ISS II: | 12 (33%) | 355 (32%) |
| ISS III: | 7 (19%) | 275 (25%) |
| Missing | 6 | 261 |
| R-ISS stage, n(%) |  |  |
| R-ISS I: | 6 (32%) | 149 (21%) |
| R-ISS II: | 11 (58%) | 469 (67%) |
| R-ISS III: | 2 (11%) | 82 (12%) |
| Missing | 23 | 654 |
| Cytogenetic risk profile, n(%) |  |  |
| Standard | 26 (93%) | 578 (80%) |
| High-risk cytogenetics^***^ | 2 (7%) | 146 (20%) |
| Missing | 14 | 630 |

*Kidney failure: Creatinine > 177 μmol/l or CrCl <40 ml/min. Anemia: Hemoglobin <10 g/dL or >2 g/dL below normal. Osteolytic lesions: one or more osteolytic lesion x-ray, CT or PET-CT. Other: IgM, IgD, non-secretory, biclonal. ASCT: autologous stem cell transplant. VCD:bortezomib, cyclophosphamide, dexamethasone. VRD: bortezomib, lenalidomide, dexamethasone.

**≥60% bone marrow plasma cells, free light chain ratio ≥100 and/or >1 MRI-defined ≥5 mm focal lesion

***High risk cytogenetics was defined as (17p-, t(4;14) or t(14;16))

****Patients who changed treatment, were excluded from this analysis

**Supplementary Table 4: Response rates, VTD induction and total ASCT-population**

|  | VTD induction (n=42) | All (n=1354) |
| --- | --- | --- |
| Before ASCT (after induction) |  |  |
| nCR/CR/sCR | 9 (39%) | 195 (18%) |
| VGPR | 11 (48%) | 399 (36%) |
| ≥VGPR | 87% | 54% |
| PR | 2 (9%) | 422 (38%) |
| Overall response (≥PR) | 96% | 92% |
| SD | 1 (4%) | 99 (9%) |
| PD | 0 | 7 (1%) |
| Excluded due to change of therapy | 16 | 105 |
| Missing | 3 | 127 |
| 3 months after ASCT |  |  |
| nCR/CR/sCR | 14 (54%) | 445 (35%) |
| VGPR | 10 (38%) | 458 (36%) |
| ≥VGPR | 92% | 71% |
| PR | 0 | 205 (16%) |
| Overall response (≥PR) | 92% | 87% |
| SD | 1 (4%) | 11 (1%) |
| PD | 0 | 38 (3%) |
| Death (from any cause) | 1 (4%) | 8 (1%) |
| Excluded due to change of therapy | 16 | 105 |
| Missing | 0 | 84 |
| Intention to treat analysis: |  |  |
| Before ASCT (after induction) |  |  |
| ≥VGPR | 25 (71%) | 625 (51%) |
| 3 months after ASCT |  |  |
| ≥VGPR | 32 (89%) | 957 (76%) |

Abbreviations: CR = complete response nCR = near complete response sCR = stringent complete respons VGPR = very good partial response PR=partial response SD = stable disease PD =progressive disease

**Supplementary Table 5: Change of therapy and reason for change of therapy, VTD and total ASCT-population**

|  | VTD induction (n=42) | All (n=1354) |
| --- | --- | --- |
| Change of therapy | 16 (38%) | 105(8%) |
| Reason for change of therapy |  |  |
| Lack of response | 0 (0% | 37 (3%) |
| Side effects | 15 (36%) | 44 (3%) |
| Progression | 1 (2%) | 19 (1%) |
| Doctor’s choice | 0 (0%) | 5 (0%) |

**Supplementary Figure 2: Kaplan-Meier Curves for PFS, VCD vs VRD in patients with high-risk cytogenetics**

**
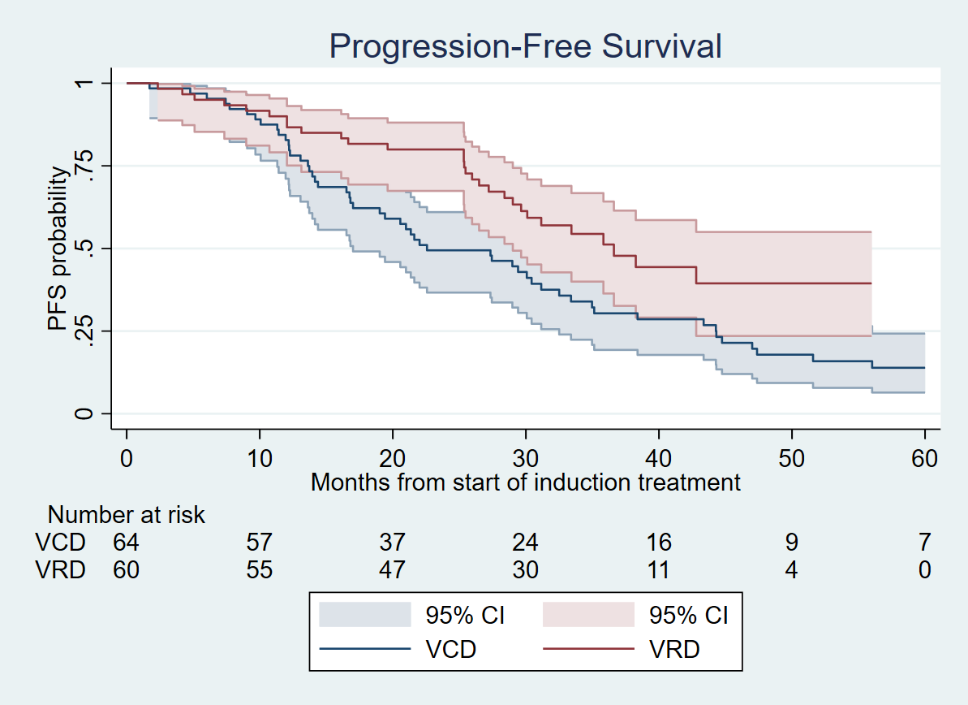
**

**Supplementary Figure 3: Kaplan-Meier Curves for PFS, VCD vs VRD in patients with standard-risk cytogenetics**

**
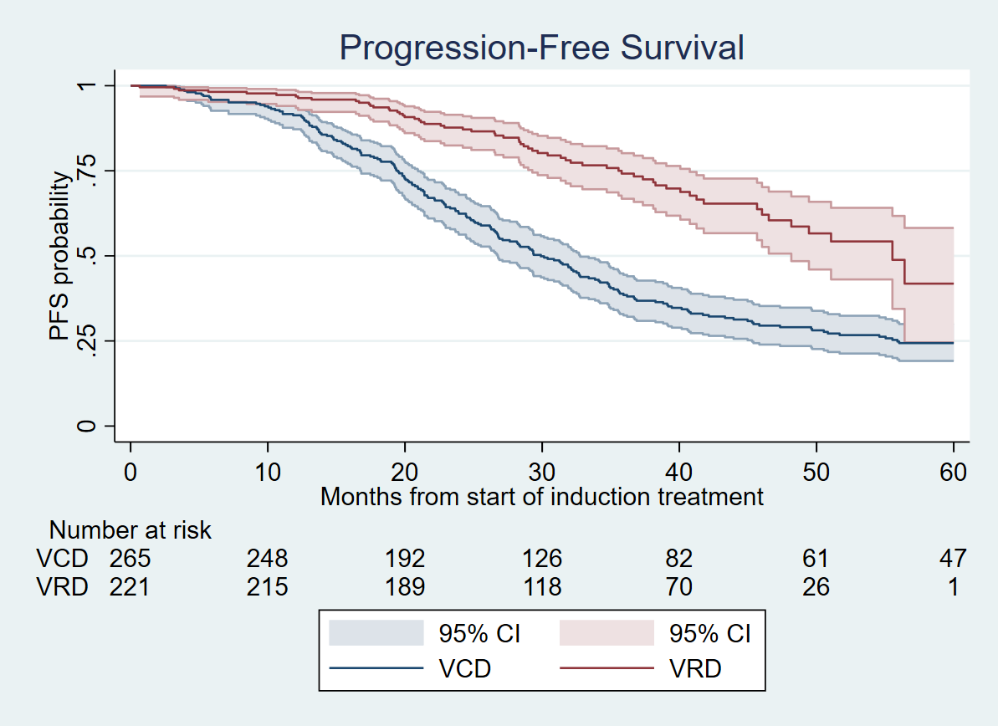
**

**Supplementary Figure 4: Kaplan-Meier curves for OS, VCD vs VRD in patients with high-risk cytogenetics**

**
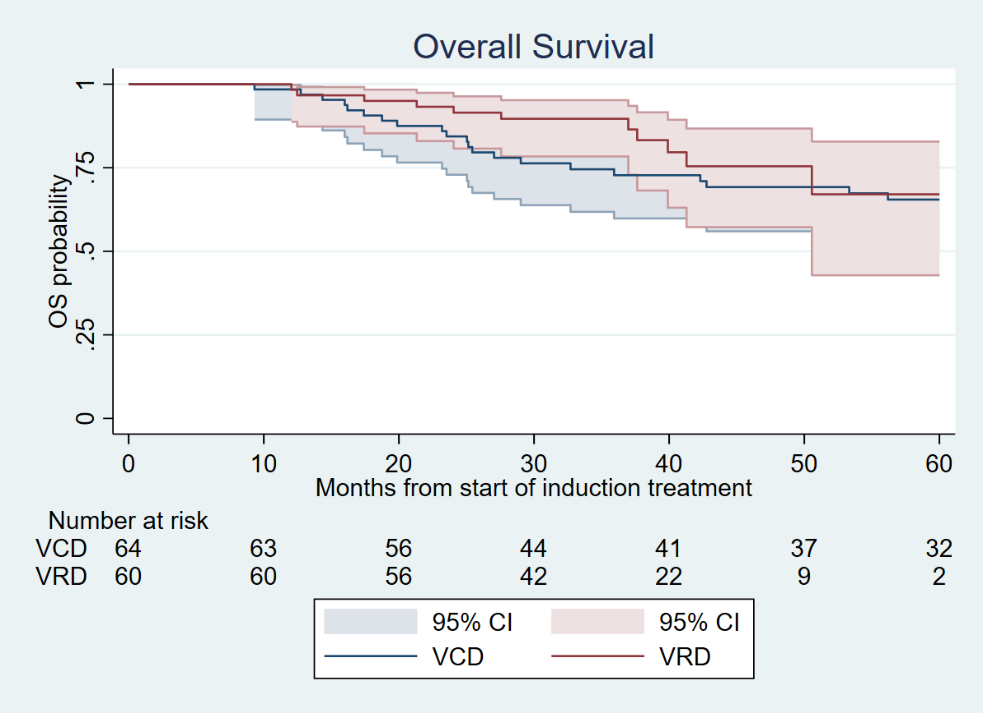
**

**Supplementary Figure 5: Kaplan-Meier Curves for OS, VCD vs VRD in patients with standard-risk cytogenetics**

**
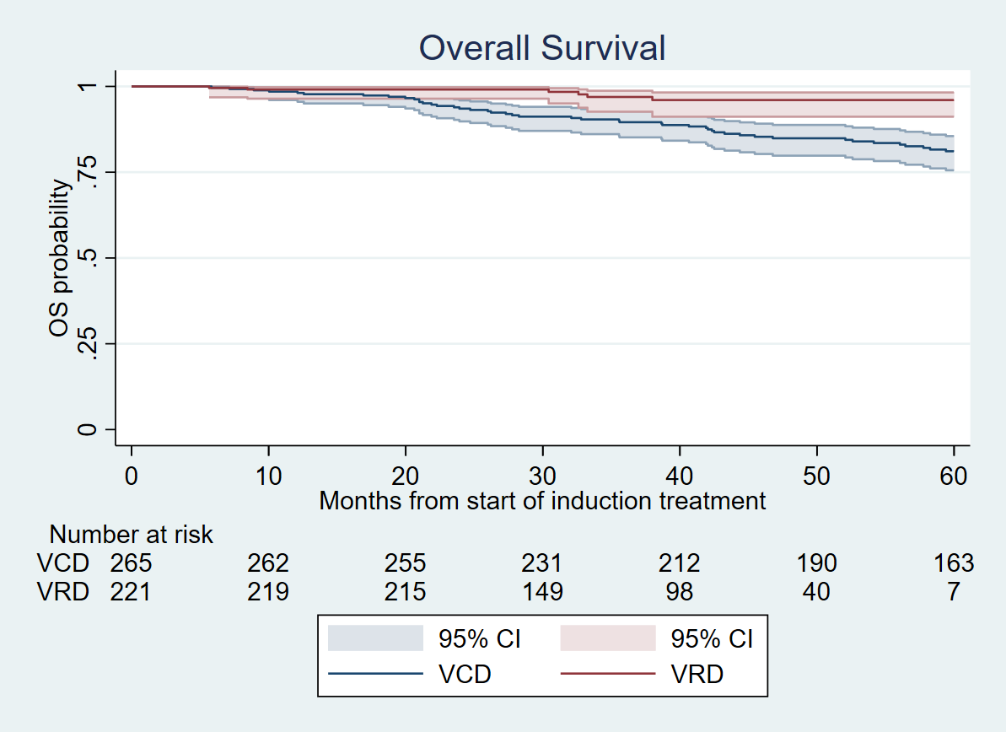
**

**Supplementary Figure 6: Kaplan-Meier Curves, OS for VCD patients by cytogenetics**

**
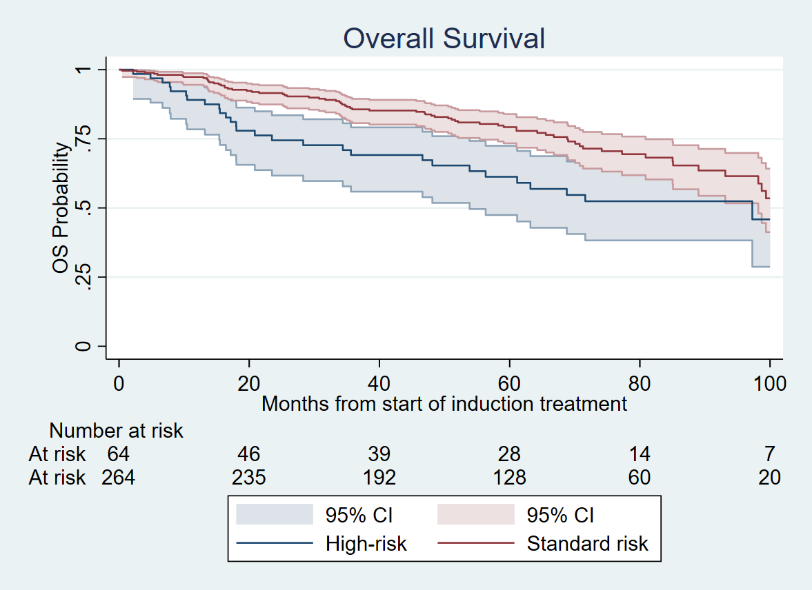
**

**Supplementary Figure 7: Kaplan-Meier Curve, OS for VRD patients by cytogenetics**

**
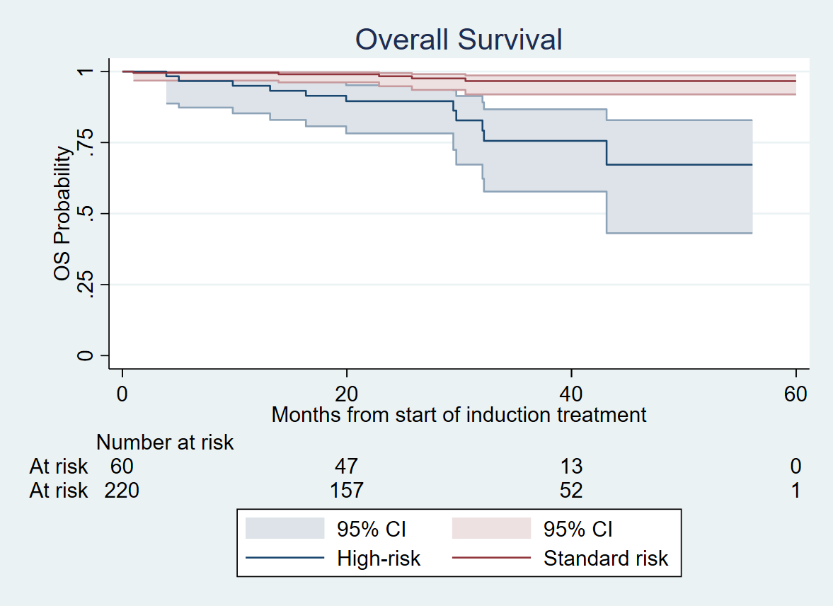
**

**Supplementary Figure 8: Kaplan-Meier Curve, PFS for VTD patients**

**
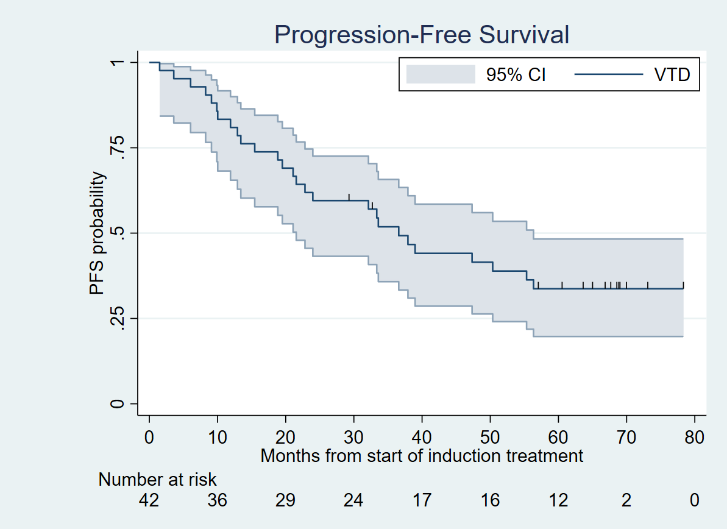
**

Median PFS: 36.6 months

**Supplementary Figure 9: Kaplan-Meier Curve, OS for VTD patients**

**
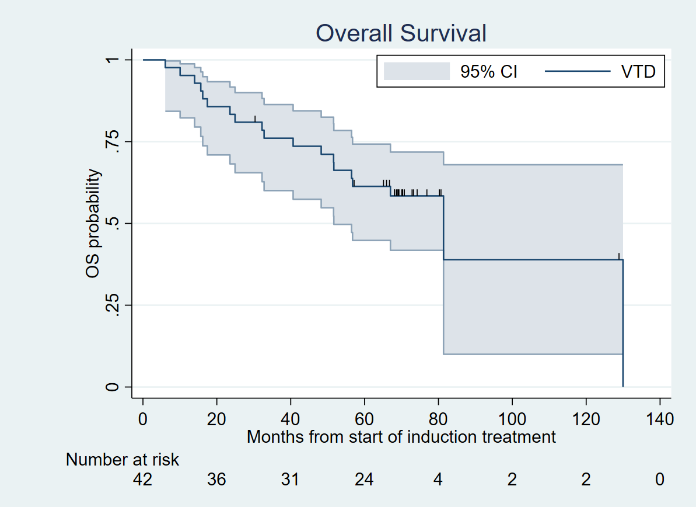
**

Median OS: 81.4 months

**Supplementary Table 6: Relevant time points and ASCT**

|  | VCD | VRD | p-value | All |
| --- | --- | --- | --- | --- |
| Number of patients with ASCT >1 year from diagnosis | 12 (2%) | 5 (2%) |  | 21 (2%) |
| Number of patients with ASCT >1 year from start of treatment | 0 (0%) | 2 (1%) |  | 5 (0.4%) |
| Diagnosis to ASCT, (median, days, range) | 149 (72-805) | 158 (113-534) | <0.001 | 151 (65-805) |
| Start of induction treatment to ASCT (median, days, range) | 134 (50-356) | 144 (101-478) | <0.001 | 137 (50-478) |
